# Multi-database bibliometric analysis and cross-database comparison of NOSES research in colorectal cancer: integrated evidence from WoSCC and PubMed

**DOI:** 10.3389/fonc.2026.1803748

**Published:** 2026-05-20

**Authors:** Siyuan Wang, Xin Liu, Boyu Kang, Yuanyao Zhang, Bo Ma, Yang Yang

**Affiliations:** 1 Department of General Surgery, The 989th Hospital of the Joint Logistics Support Force of People’s Liberation Army, Luoyang, Henan, China; 2 Department of Digestive Surgery, Xijing Hospital of Digestive Diseases, Fourth Military Medical University, Xi’an, Shaanxi, China; 3 Department of Nutrition, The 989th Hospital of the Joint Logistics Support Force of People’s Liberation Army (PLA), Luoyang, Henan, China

**Keywords:** Bibliometrix, CiteSpace, colorectal cancer, natural orifice specimen extraction surgery (NOSES), VOSviewer

## Abstract

**Background:**

As an innovative surgical approach in the treatment of colorectal cancer, Natural Orifice Specimen Extraction Surgery (NOSES) offers several advantages. To ensure robust scientific comparison through multi-database inquiry, this study conducted a comprehensive bibliometric analysis using dual independent databases to explore research hotspots and future trends in colorectal cancer NOSES.

**Methods:**

This study employed a cross-database comparison strategy using two independent sources: 432 documents from the Web of Science Core Collection (WoSCC) and 411 documents from PubMed, covering the period from January 2010 to June 2025. The WoSCC dataset served as the primary analysis cohort to establish research trends, while the PubMed database functioned as an independent validation cohort to confirm and cross-verify key findings. Bibliometric analyses of authors, institutions, countries, publishing journals, abstracts, keywords, and cited documents were performed using the Bibliometrix R package, CiteSpace, and VOSviewer. Comparative analyses between the two databases were conducted to ensure robustness and reliability of identified research patterns.

**Results:**

The analysis included articles from 576 institutions, 112 journals, and 49 countries. China led global publications with 273 articles, while the United Kingdom achieved the highest citation count (2,306). Surgical Endoscopy emerged as the most prolific journal (46 papers) and the most cited (1,824 citations). Highly cited works predominantly focused on clinical trials and innovative surgical techniques in NOSES for colorectal cancer. Cross-database comparison confirmed that recent key research themes include “orifice specimen extraction,” “chemoradiotherapy,” and “pathological outcomes,” reflecting the field’s evolving priorities toward minimally invasive approaches and multidisciplinary integration.

**Conclusion:**

This study reviewed the research on NOSES for colorectal cancer, providing a reference for subsequent studies. It mapped the research landscape in this field, summarized the current state of research, and offered data support and insights for future development.

## Introduction

The incidence of colorectal cancer is on the rise, imposing a substantial burden on health and the economy (1). Despite significant advancements in immunotherapy and targeted therapy, surgery remains the primary therapeutic strategy (2). To reduce surgical trauma and improve patient prognosis, laparoscopic surgery was introduced into the treatment strategy for colorectal cancer in 1991, demonstrating significant advantages (3, 4). Compared with traditional open surgery, laparoscopic surgery has advantages in reducing postoperative pain and shortening recovery time. It also demonstrated non-inferiority to traditional surgery in terms of safety, margin status, and completeness of resection (5). Follow-up studies further suggest that laparoscopic surgery has a better three-year disease-free survival rate, highlighting the development of minimally invasive surgery and its rise to prominence (6). In recent years, as a new mode of laparoscopic surgery, robotic surgery has improved surgical quality with its three-dimensional vision and flexible robotic arms (7). However, robotic surgery has not significantly reduced the number of surgical incisions.

Recent years, clinicians have increasingly sought to reduce the size and number of surgical incisions (8). Consequently, NOSES emerged, performing surgeries through the body’s natural orifices to minimize surgical incisions and protect abdominal nerves and other tissues (9). NOSES is one of the least invasive options for treating colorectal cancer and helps reduce postoperative complications, which may be related to the avoidance of auxiliary abdominal incisions (7). The current international consensus holds that NOSES primarily encompasses surgical procedures such as natural orifice specimen extraction surgery, natural orifice transluminal endoscopic surgery (NOTES), and transanal total mesorectal excision (taTME), which involve specimen extraction through natural orifices (10). In specific patients, these methods can serve as alternatives to traditional open surgery and laparoscopic surgery (11).

Bibliometric analysis provides high-quality visual maps to clarify the current state and future trends of NOSES research (12). By quantifying and interpreting a large number of previous studies, bibliometric analysis elucidates current research achievements and predicts future development trends, offering researchers scientifically robust and referential guidance (13).

This study, based on the thriving trend of minimally invasive surgery and the current exploratory status of NOSES, evaluates research achievements in this field over the past 15 years through bibliometric analysis, rather than a traditional narrative review, to gather valuable insights that have a profound impact on clinical practice. Specifically, this work addresses several key research questions, including the temporal trends, geographic distribution, and international collaboration patterns of colorectal NOSES research, the contributions of major authors, institutions, countries, and journals, the identification of research hotspots and frontiers, and the consistency and discrepancies between WoSCC and PubMed databases. Therefore, for clinicians seeking to master the latest evidence-based interventions and maintain consistency, as well as for researchers eager to fill knowledge gaps and extend into new areas, bibliometric maps are indispensable. We have also provided a structured synthesis of the principal results to enhance the logical coherence of the manuscript. Essentially, this study aims to serve as a cornerstone for optimizing patient outcomes and guiding future research and exploration in the context of colorectal cancer NOSES. We declare that this study is based on the TITAN guidelines and transparently reports the use of AI in any manuscript (14).

## Materials and methods

### Retrieval strategy and data collection

**Figure 1:**
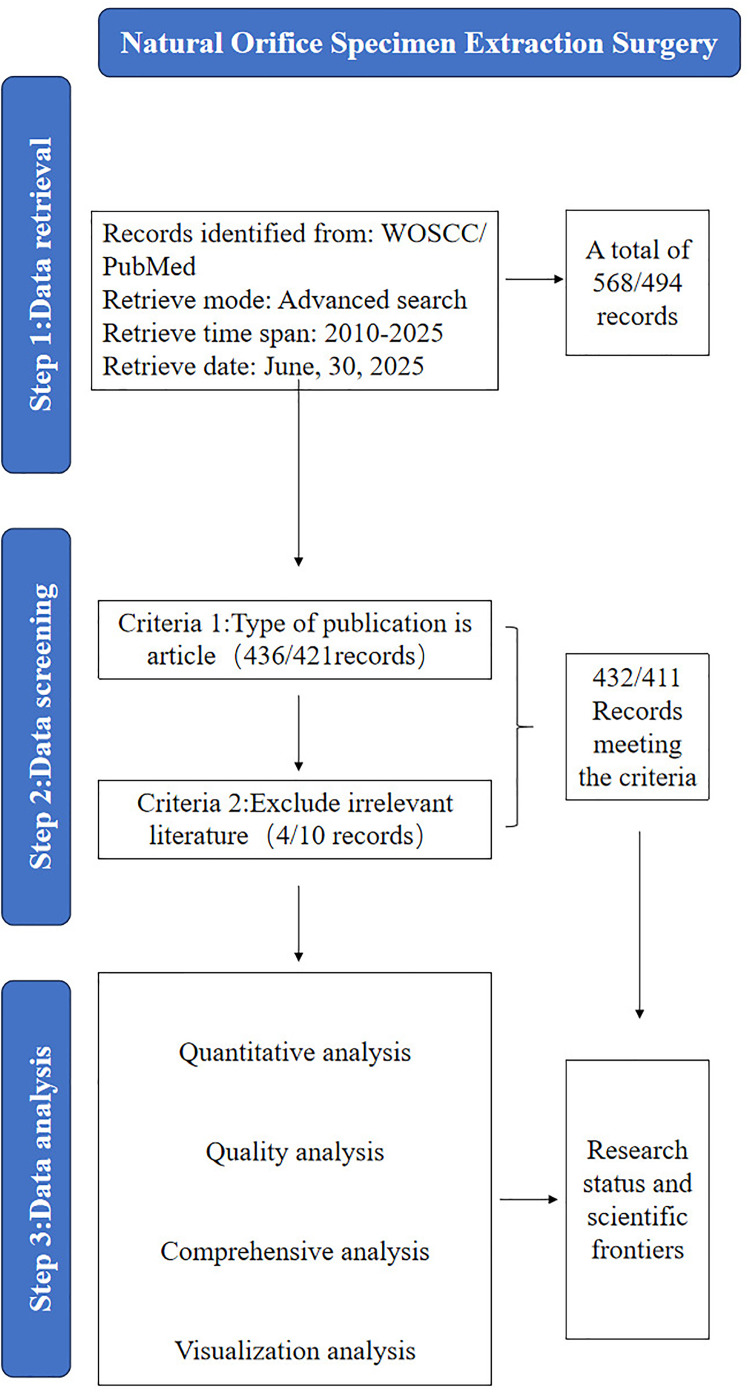
Flow chart of literature selection.

We chose Web of Science Core Collection (WoSCC) and PubMed as the source database for data retrieval in this study. The literature search in the Web of Science Core Collection (WoSCC) was performed with the following topic (ts) retrieval strategies: #1, ((((ts= (CRC)) or ts= (colorectal neoplasia)) or ts= (colorectal tumor)) or ts= (colorectal cancer)) or ts= (colorectal carcinoma) #2, (((ts=(natural orifice specimen extraction surgery)) OR ts=(Natural orifice translumenal endoscopic surgery)) OR ts=(transanal total mesorectal excision)) OR ts=(taTME), #1 AND #2. For the PubMed database, the search strategy was constructed as follows: #1, (“colorectal cancer”[Title/Abstract]) OR (“colorectal carcinoma”[Title/Abstract]) OR (“colorectal neoplasia”[Title/Abstract]) OR (“colorectal tumor”[Title/Abstract]) OR CRC[Title/Abstract]; #2, (“natural orifice specimen extraction surgery”[Title/Abstract]) OR (“natural orifice translumenal endoscopic surgery”[Title/Abstract]) OR (“transanal total mesorectal excision”[Title/Abstract]) OR taTME[Title/Abstract];The final retrieval formula was set as #1 AND #2. The search period was set from January 1, 2010, to January 1, 2025, and the material type was limited to monographs. The search date for this study was June 30, 2025. The language of the studies was restricted to English. All duplicate records retrieved from different databases were firstly identified and removed using the built-in deduplication function of EndNote. After automatic deduplication, we further manually screened the remaining literatures to check and eliminate highly repetitive articles with identical titles, authors and publication years, so as to ensure the uniqueness of all included records. After excluding irrelevant literatures, 432 articles and 411 articles met the inclusion criteria. The flow chart of literature screening is shown in **Figure 1**.

### Data extraction and bibliometric analysis

We selected the articles published within the past 15 year. We employed bibliometric analysis algorithm to identify the references with the highest citation explosion point, keyword explosion point and citation, keyword clustering, so as to reveal the research hotspots and trends in the field of NOSES in colorectal cancer.

The bibliometric and visualization tools used in this paper were VOSviewer (version 1.6.20, Leiden University, the Netherlands), CiteSpace (version 6.3.R1, Drexel University, Pennsylvania, USA), and the R studio (version 4.3.0) programming language. VOSviewer constructs bibliometric collaboration networks based on co-authorship, citation, and bibliographic coupling data, covering aspects such as authors, institutions, journals, and keywords. CiteSpace analyzes keyword and citation bursts within specific time periods, which helps to identify research trends and hotspots over time. The Bibliometrix package in the R studio serves as a fundamental platform for bibliometric analysis, visualizing data on a macro level. It prepares data for further visualization and interpretation using the aforementioned tools through initial quantitative analysis. The content of the analysis mainly included countries, institutions, authors, journals, references and keywords. Data were stored and processed in txt format.

### Parameter setting

After importing the documents into CiteSpace, the scaling factor k was adjusted to 18–25 to obtain appropriate g values and network sizes. The settings were modified to analyze author, keyword, and citation nodes, with a focus on the top 25 most cited or mentioned items each year. The top 10 items were extracted for visualization, while the remaining items were saved in tabular form. Burst detection analysis was then performed after processing the documents. In VOSviewer, data related to co-authorship and keyword co-occurrence were processed by setting thresholds to ensure the inclusion of the most relevant and impactful data, typically ranging from 40 to 60 documents. The results were manually fine-tuned to provide clear, aesthetically pleasing, and scientifically sound visualizations.

## Result

### Trends in publications of studies related to NOSES in colorectal cancer

**Figure 2:**
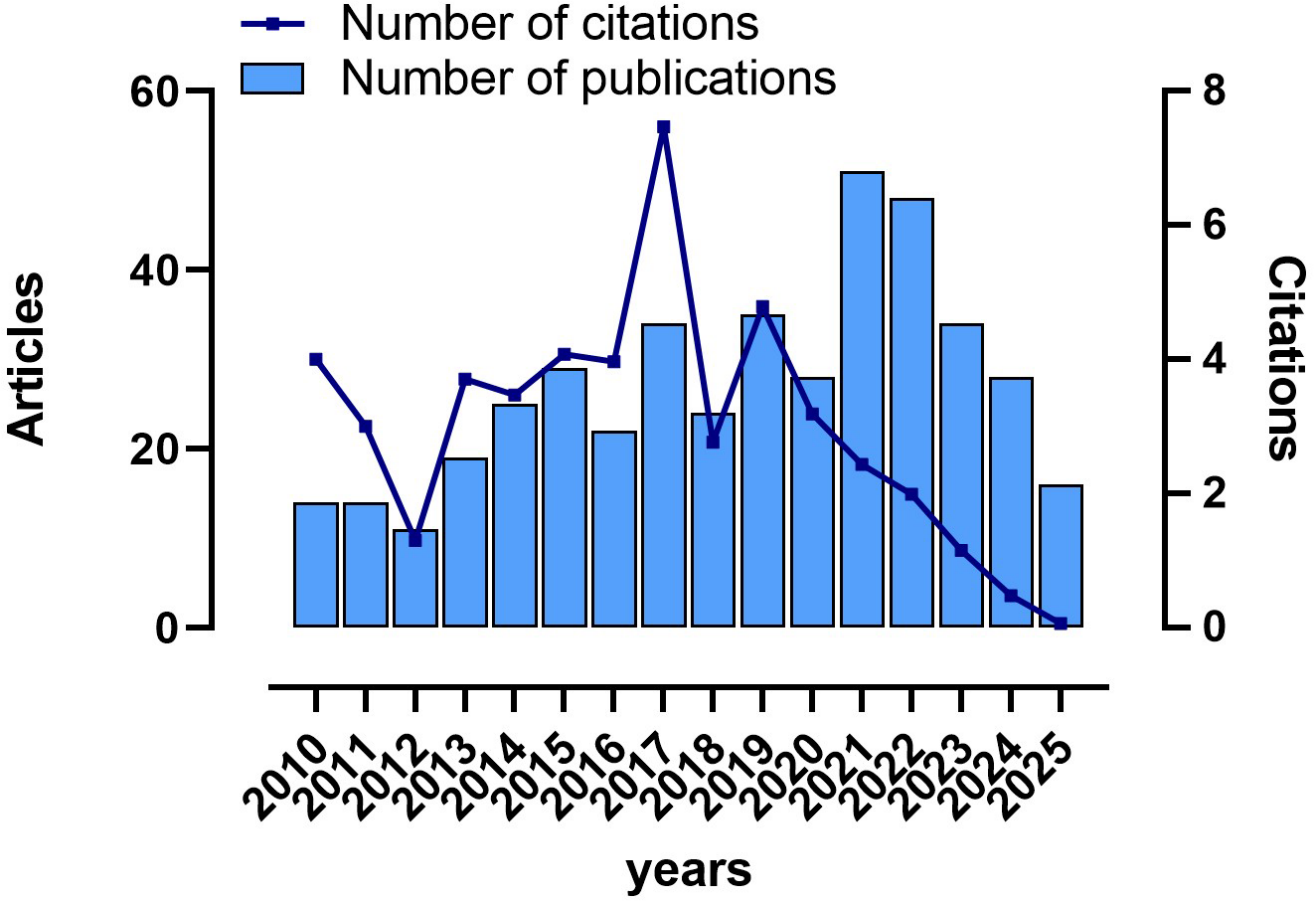
Trends in annual publications on NOSES in colorectal cancer.

The analysis incorporated 432 articles identified from the Web of Science Core Collection (WoSCC) database from 2010 to 2025, together with 411 articles retrieved from PubMed. A total of 112 reviews, meta-analyses, and expert consensus articles, 18 conference papers, and 2 early access articles were excluded. The total number of citations was 6480. It outlined the trends in the annual number of publications and citation frequency for NOSES (**Figure 2**). There were more publications from 2021 to 2024, and the highest average citation count was in 2017. Overall, research on NOSES was limited with large fluctuations in quantity. However, in the past five years, NOSES has emerged as a research hotspot, with an increase in the number of studies and average citation counts.

### Author output, collaboration, and citation analysis

**Figure 3:**
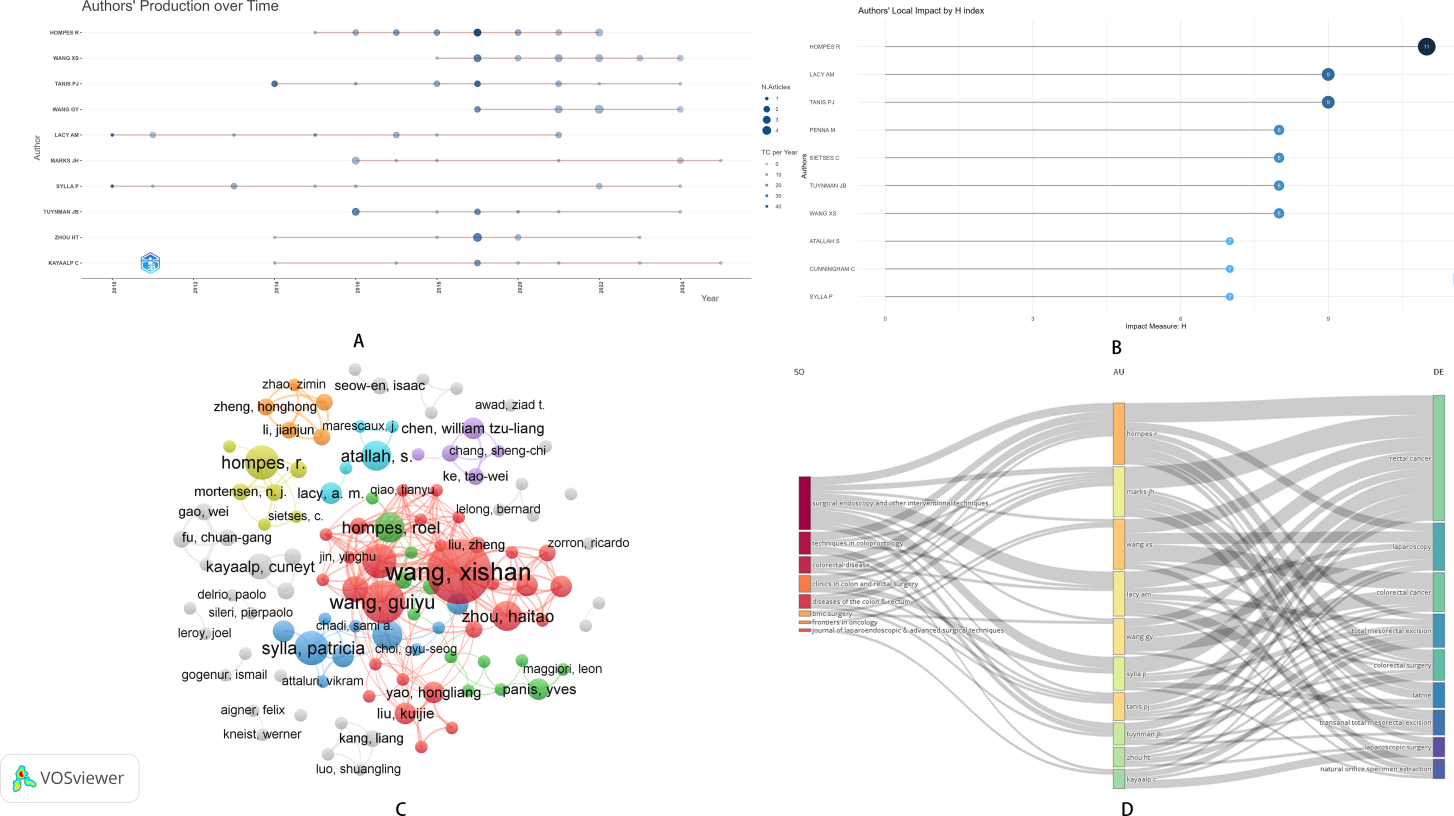
NOSES in colorectal cancer. **(A)** Time chart of author output. **(B)** The H-index of authors. **(C)** Co-citation chart of authors. **(D)** Sankey Diagram of journals, authors, and keywords.

A total of 2,328 authors have contributed to the research on NOSES in colorectal cancer. Between 2010 and 2025, HOMPES R. published the most research outputs, which were mainly concentrated from 2015 to 2022. SYLLA P. had the longest research span, but the number of research output has declined in recent years (**Figure 3A**). In terms of influence, HOMPES R. was the most influential scholar, with an H-index of 11 (**Figure 3B**). Regarding author collaboration, Chinese authors have collaborated the most in the field of NOSES in colorectal cancer. Wang Xishan from the Chinese Academy of Medical Sciences had the highest number of author collaborations, followed by Professor Wang Guiyu from Harbin Medical University (**Figure 3C**). The Sankey diagram illustrated the connections between journals, authors, and keywords. Professor HOMPES R. has published research in multiple journals, including Surgical Endoscopy and Other Interventional Techniques, with keywords related to “rectal cancer” and “laparoscopy” (**Figure 3D**). Among the top 10 authors ranked by H-index, HOMPES R. had a relatively high H-index and the highest average citation count (**Table 1**). Analysis based on the PubMed database demonstrated that R. HOMPES exhibits outstanding academic influence. Regarding author collaboration, Chinese scholars such as Kang Liang show significant collaborative activity; however, scholars including R. HOMPES also maintain extensive author collaboration networks (**Supplementary Figure 1**).

**Table 1:** Author basic information in colorectal cancer NOSES.

Author	H index	G index	M index	TC	NP	PY start
HOMPES R	**11**	**17**	**1**	848	**17**	2015
LACY AM	9	10	0.563	**1052**	10	2010
TANIS PJ	9	11	0.75	829	11	2014
PENNA M	8	8	0.8	642	8	2016
SIETSES C	8	8	0.615	617	8	2013
TUYNMAN JB	8	9	0.8	487	9	2016
WANG XS	8	16	**1**	279	16	2018
ATALLAH S	7	7	0.583	273	7	2014
CUNNINGHAM C	7	7	0.636	221	7	2015
SYLLA P	7	9	0.438	798	9	2010

TC, Total Citations; NP, Number of Publications; PY_start, Publication Year Start.

Bolded text indicates the maximum value in this column.

### Country/region and institution contributions analysis

**Figure 4:**
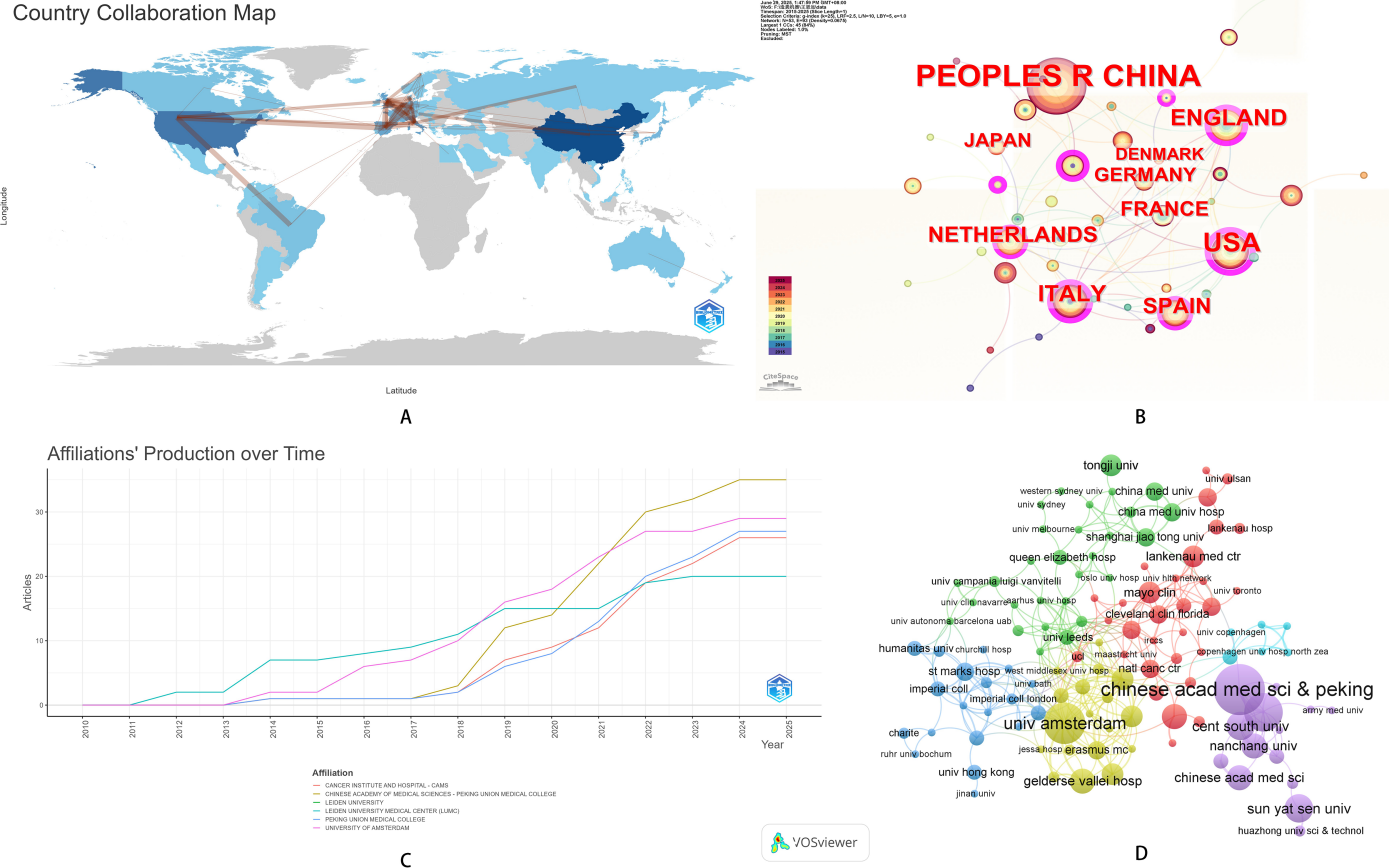
Country/region and institution contributions analysis. **(A)** National document volume map of neoadjuvant therapy for colorectal cancer. **(B)** National cooperation map. **(C)** Institutional document trend map. **(D)** Institutional interaction map.

In terms of national publication counts, the top three countries were China, the United States, and Netherlands. It was particularly noteworthy that both the United States and China had a high number of total citations but a relatively low average citation per document. This highlighted the urgency of improving article quality. Compared with other countries, the United States and China had close connections in the collaboration network, which may be one of the reasons for the high number of research outputs from these two countries (**Figure 4A**, **Table 2**). As of 2025, we identified the top 10 countries by NOSES publication output (**Figure 4B**). Among these nations, only China is classified as a developing country. This finding suggests that the solid economic foundation of developed countries may facilitate advances in cutting-edge surgical research. University of Amsterdam and Peking Union Medical College were well-known for their research on NOSES. However, in 2021, The Chinese Academy of Medical Sciences in collaboration with Peking Union Medical College became the institution with the highest number of publications in this field (**Figure 4C**). In terms of institutional collaboration, University of Amsterdam has collaborated more with Gelderse Vallei Hospital, while Peking Union Medical College had close ties with Nanchang University, Sun Yat-sen University, and other institutions, indicating that institutional collaboration had a distinct regional nature (**Figure 4D**). Findings from the PubMed database were consistent with those from the Web of Science Core Collection (WoSCC), jointly demonstrating the substantial influence and extensive collaboration of the United States, China, and the Netherlands (**Supplementary Figure 2**).

**Table 2:** Centrality of national cooperation.

Country	Cluster	Betweenness	Closeness	PageRank
USA	2	**260.042**	0.015	0.068
Italy	3	222.925	**0.017**	0.079
China	1	121.579	0.014	0.041
United Kingdom	3	106.187	0.016	**0.084**
Spain	3	73.61	0.014	0.061
Korea	1	50.094	0.014	0.028
Turkey	1	42	0.012	0.021
France	3	16.929	0.014	0.033
Norway	3	16.014	0.013	0.028
Egypt	1	11.378	0.013	0.022
Germany	3	10.066	0.013	0.042
Japan	1	8.035	0.013	0.027
Portugal	1	6.512	0.013	0.025
Canada	2	6.453	0.011	0.016
Netherlands	3	5.734	0.013	0.053
Brazil	2	0.889	0.011	0.019
Belgium	3	0.543	0.012	0.019
Denmark	3	0.278	0.011	0.027

The bold values are used to highlight the highest (or most prominent) values within each respective centrality column, drawing attention to the top-performing countries for that specific metric. Specifically: USA (260.042) have the highest Betweenness scores, indicating that they serve as the most critical intermediaries or bridges in the cooperation network. Italy (0.017) has the highest Closeness score, meaning it is the most centrally positioned and can reach other countries most efficiently. United Kingdom (0.084) has the highest PageRank score, suggesting it holds the greatest influence or prestige in the network.

### Journal publications analysis

**Figure 5:**
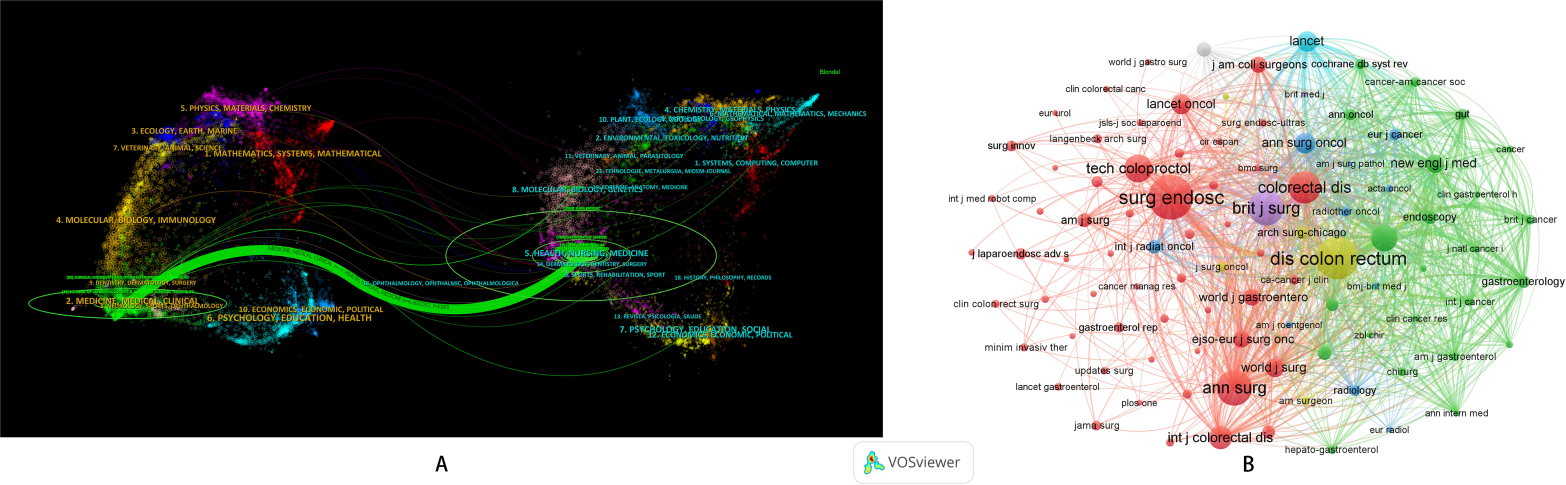
NOSES in colorectal cancer. **(A)** Dual map overlay. **(B)** Journal co-cited.

According to the analysis by Bibliometrix, the journal with the highest number of publications was *Surgical Endoscopy and Other Interventional Techniques*. We adopted the dual-map overlay analysis in CiteSpace. Citation-link curves were applied to connect cited journals on both sides. In this visualization, the ellipse length indicates the author quantity, while the ellipse width reflects the publication number. Our research findings indicated that medical and clinical medicine were influenced by health medicine and nursing (**Figure 5A**). This suggested the existence of interdisciplinary intersections in the field, including clinical medicine, public health, nursing, and other social sciences. Bradford’s Law helped further identify core journals within the field, which can guide researchers in their literature search and manuscript submission (**Supplementary Figure 1**). Co-cited journals showed that, in addition to journals with a high number of publications, well-known industry journals such as *Diseases of the Colon & Rectum* also had a high number of co-citations (**Figure 5B**). The PubMed database also highlighted the prominent status of *Surgical Endoscopy and Other Interventional Techniques* and identified *Techniques in Coloproctology* as the second most influential journal in the field of NOSES (**Supplementary Figure 3**).

### Analysis of co-citation reference and reference citation bursts

**Figure 6:**
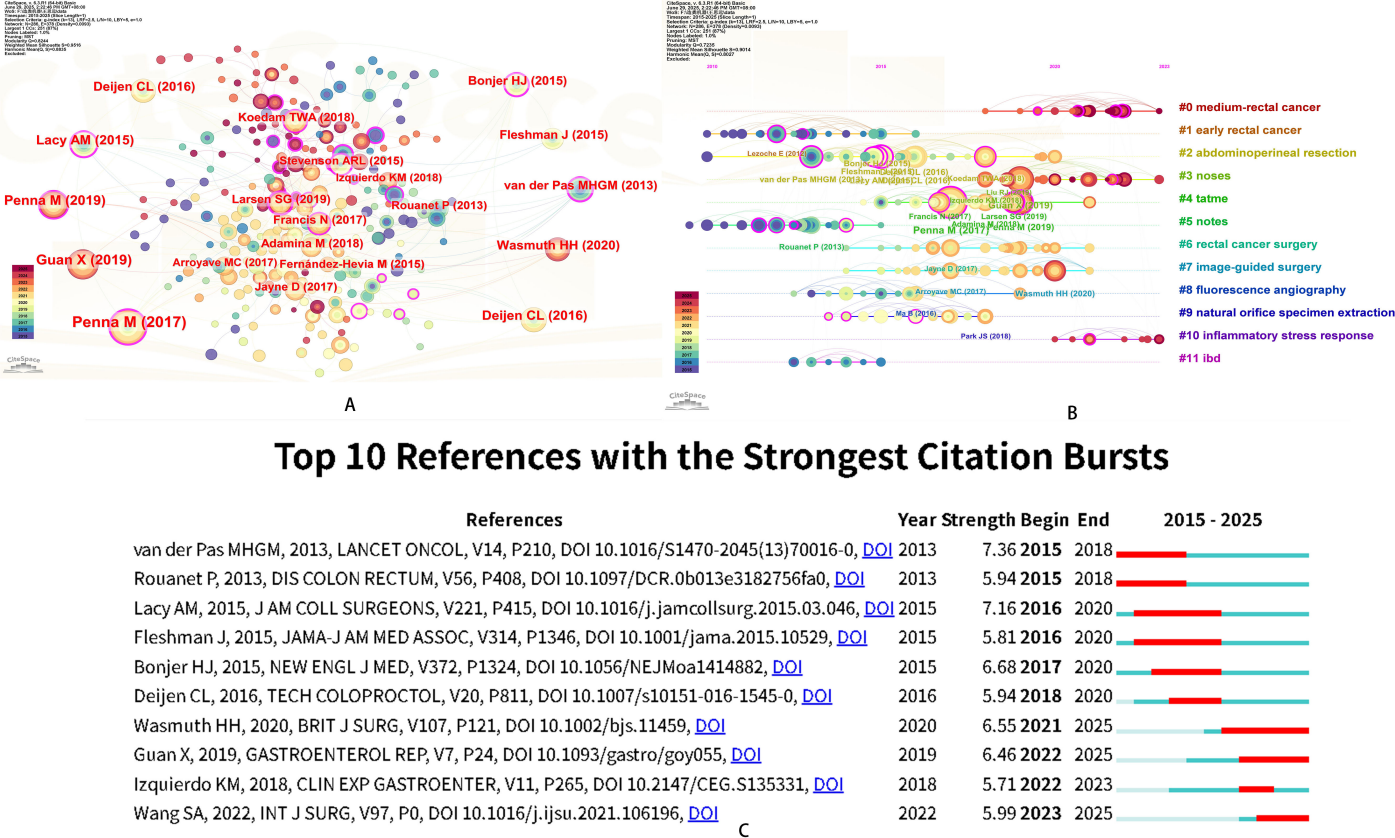
Reference analysis of NOSES in colorectal cancer. **(A)** Co-cited literature interaction. **(B)** Co cited literature label clustering analysis. **(C)** Burst analysis of co-citation literature.

Through co-citation analysis, the research hotspots and frontiers in this field were revealed. In the co-citation network map, different cited document nodes were connected by lines, indicating that they have been cited in the same publication. Co-citation clustering analysis and timeline analysis were performed using CiteSpace, and the top 10 most cited documents were analyzed (**Figure 6A**). A key study was conducted by Marta Penna and published in *Annals of Surgery* in 2017. This research collected clinical data from 66 medical centers worldwide. The findings demonstrated that NOSES is a safe and effective technique for oncologically radical distal rectal mesorectal dissection. It yields satisfactory short-term postoperative outcomes and reliable surgical specimen quality (15). This surgical approach may offer new insights for improving patient prognosis. The timeline analysis of co-cited documents categorized the documents into eleven clusters, with circles on each track representing the time of document appearance (**Figure 6B**). To elucidate high-frequency keywords that burst at specific moments, burst detection analysis of co-cited documents was conducted, highlighting research hotspots in the field and indicating future research trends. The duration of co-cited documents was shown in red (**Figure 6C**). The results indicated that from 2010 to 2025, surgical treatment for rectal cancer has consistently been a research hotspot in the field of colorectal cancer NOSES. The results of co-citation detection analysis revealed that mid-rectal cancer has emerged as a focal point of research since 2018. This trend aligned with the burgeoning development of NOSES over the past several years, thereby highlighting the relatively nascent nature of research within the tumor NOSES domain. In the realm of basic research, the inflammatory stress response associated with NOSES was likely to represent an emerging area of interest for future investigation. Co-citation analysis in the PubMed database showed that dMMR/MSI-H colorectal cancer patients and tumor-associated macrophages represent current research focuses, which also clarified the important value of adjuvant therapy (**Supplementary Figure 4**).

### Keyword co-occurrence and burst analysis

**Figure 7:**
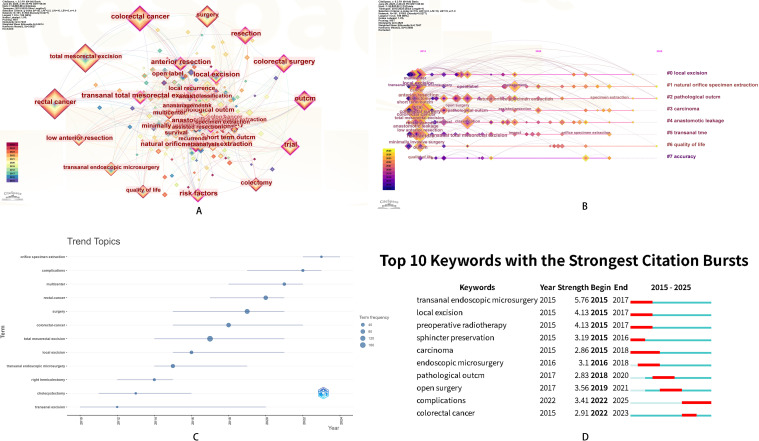
NOSES in colorectal cancer. **(A)** Keyword co-occurrence **(B)** Keyword temporal analysis **(C)** Keyword trend topic **(D)** Keyword burst analysis.

**Figure 8:**
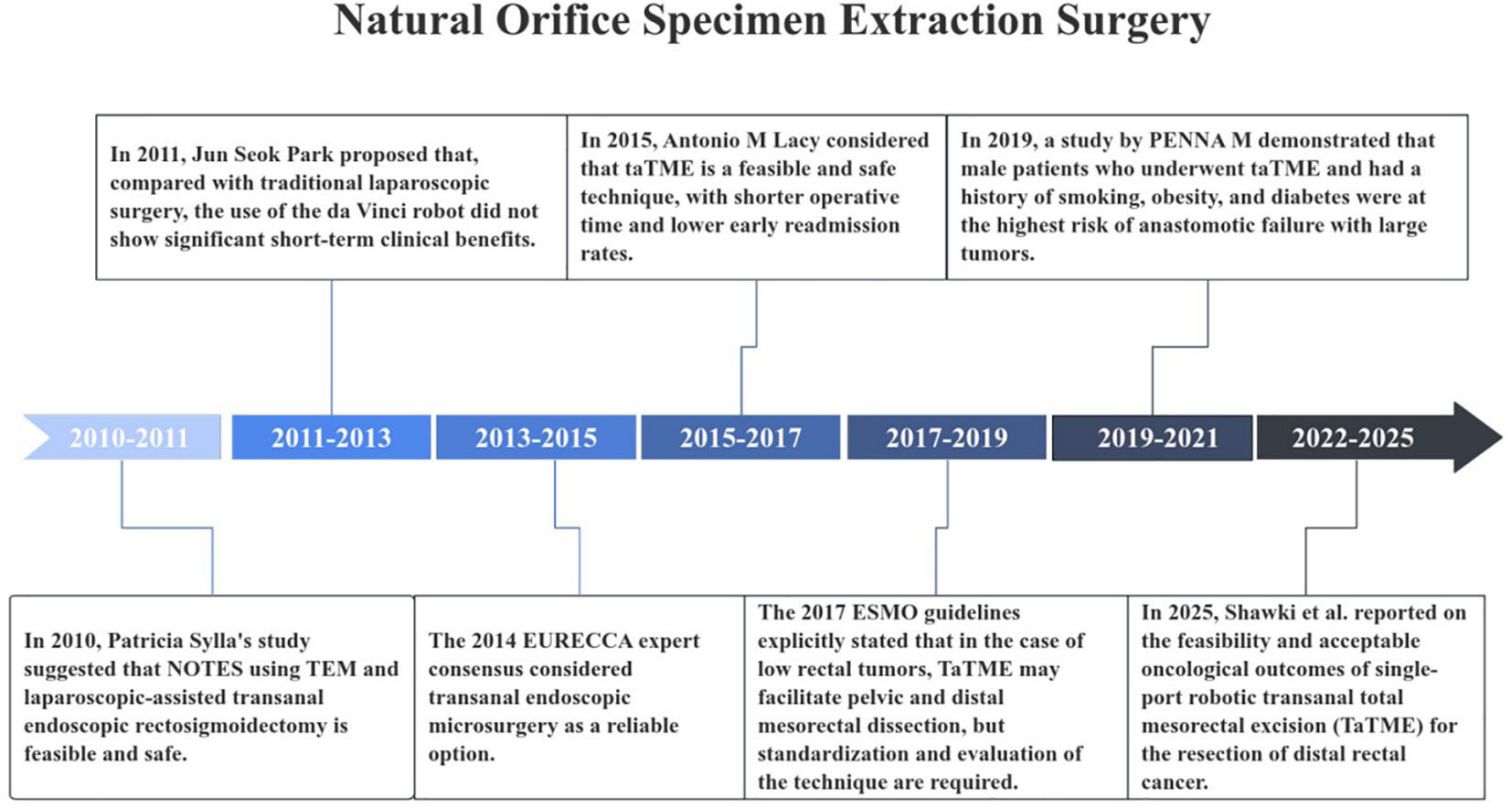
Important milestones in NOSES in colorectal cancer.

Through keyword co-occurrence analysis, we identified 78 keywords that appeared three or more times from the 743 keywords included in the dataset. Among them, “rectal cancer” was the most frequently occurring keyword. In the keyword clustering analysis, we divided the keywords into eight clusters and performed time-series analysis (**Figure 7A**). The timeline from 2010 to 2024, combined with the time-series analysis of keywords and trend topics, highlighted research progress in this field (**Figures 7B, C**). In recent years, research in this area has mainly focused on “orifice specimen extraction”, “quality of life” and “pathological outcomes”. It also predicted that future research directions were expected to be related to “orifice specimen extraction” and “complications”. In summary, analyzing keyword co-occurrence, bursts, and time-series in the temporal dimension highlighted the complexity of the colorectal cancer NOSES field, which encompassed multiple disciplines, including basic medicine, surgical specialties, obstetrics and gynecology, computer science, and social sciences. Therefore, interdisciplinary collaboration is crucial for the development of this field (**Figure 8D**). In the keyword analysis, the PubMed database also showed results consistent with those from the WoSCC (**Supplementary Figure 5**). Colorectal cancer and laparoscopic total mesorectal excision represented current research hotspots and future research trends.

## Discussion

### Summary of main findings

Bibliometric analysis enables us to delineate the developmental trajectory and current research landscape of this field, as well as to forecast future research trends. By conducting literature searches and downloads via WoSCC and PubMed, we employed three distinct software packages, Bibliometrix R package, CiteSpace, and VOSviewer to conduct qualitative and quantitative analyses of research in the field of colorectal cancer NOSES. Over the past 15 years, interest in the field of colorectal cancer NOSES has gradually increased, with a growing number of related studies. The rapid increase in NOSES publications after 2020 stemmed from the gradual maturation of surgical techniques and high−level clinical evidence. Accumulated multicenter data and standardized procedural systems further verified the oncological safety and clinical feasibility of NOSES, greatly promoting its clinical application (16). Meanwhile, improved academic consensus, specialized training programs, and updated clinical guidelines accelerated technical popularization and collaborative research, thereby continuously expanding related research output (10). The average citation count peaked in 2017, which may be related to a smaller number of publications causing an artificially inflated average citation count. The marked increase in the number of studies within this field over the past five years, in conjunction with relatively low citation counts, suggested that the field of NOSES for colorectal cancer was currently an emerging research hotspot. Consequently, there was a compelling necessity for additional high-quality research to further elucidate the clinical significance and optimize the application of this technique.

In the analysis of countries and institutions, we included 49 countries and 576 institutions in our study. China had the highest number of publications, and two of the top three institutions in terms of publication volume were from China. However, the overall citation count was relatively low, indicating that improving research quality is of paramount importance. The United Kingdom had the highest total number of citations, suggesting that its research is highly recognized in the field. In this field, the United States and the Netherlands are in a leading position, with a high number of publications and citations.

Dr. Roel Hompes from Amsterdam UMC is the most influential scholar in the field of colorectal cancer NOSES, with research outputs mainly concentrated between 2015 and 2022. However, he has relatively few collaborations with other authors, and these are mostly within Europe. Xishan Wang has the most extensive collaborations, working with scholars from China, South Korea, Japan, and other countries, but has fewer collaborations with scholars from Europe and the Americas. Currently, author collaboration in the field of colorectal cancer NOSES is geographically oriented, with more domestic cooperation and less international collaboration. As a new surgical technique, widespread application across different regions and ethnicities is of great significance for future promotion and the exploration of indications and contraindications. Therefore, international cooperation should be further strengthened. Nevertheless, global collaboration in NOSES research faces notable barriers. These primarily include regional disparities in surgical proficiency, inconsistent operative protocols, and uneven healthcare resource distribution worldwide. Furthermore, divergences in clinical reimbursement regulations, ethical review requirements, and insufficient multicenter coordination collectively restrict transnational data sharing and joint research initiatives.

In terms of journals, by applying Bradford’s Law to identify core journals, *SURGICAL ENDOSCOPY* is recognized as the most important core journal in the field of colorectal cancer NOSES, having published a total of 46 studies and being cited 1,824 times. It is the most cited journal, indicating its significant recognition within the field. In addition, top-tier medical journals such as *The Lancet* have also published a considerable number of research findings, reflecting the attention of leading scholars in the industry towards the field of colorectal cancer NOSES. Although China is the country with the highest number of published studies, none of the top 10 most cited journals are published in China, highlighting the urgency of establishing influential journals.

Co-citation analysis of documents and keywords reveals research hotspots in this field. The article titled “Rectal cancer: ESMO Clinical Practice Guidelines for diagnosis, treatment and follow-up,” published in *Annals of Oncology* in 2017, elaborated that NOSES may bring benefits in low rectal cancer, prompting scholars to conduct further in-depth research in this area (17). The temporal trend analysis of keywords reveals the evolution of research hotspots in this field. Initially, keywords such as “transanal endoscopic microsurgery” and “local excision” were prevalent. By 2025, the focus had shifted to keywords like “complications” and “pathological outcomes.” This shift indicates that research hotspots have transitioned from the initial exploration of surgical techniques to the current emphasis on safety and effectiveness analysis.

### Continuous updates in NOSES for colorectal cancer

The application of NOSES in colorectal cancer has gradually progressed over these 15 years. Dividing the period from 2010 to 2025 every five years, the developmental trajectory of NOSES for colorectal cancer was delineated into three stages, each characterized by the exploration of advantages, the establishment of guidelines and standards, and the integration of multidisciplinary approaches. From 2010 to 2015, NOSES for colorectal cancer was in the phase of exploring its advantages. Researchers explored the significant advantages of NOSES over laparoscopic and open surgery through clinical studies. In 2010, SYLLA P. reported the first clinical case of treating rectal cancer with TEM and laparoscopic-assisted transanal resection in *Surg Endosc*, first proving that natural orifice translumenal endoscopic surgery for rectosigmoidectomy was feasible and safe (18). In 2014, the EURECCA European Consensus Conference first proposed that transanal endoscopic microsurgery could be a reliable option for treating rectal cancer through an expert consensus meeting (19). This proposal sparked widespread interest among scholars in the field of colorectal cancer NOSES, prompting an increasing number of researchers to explore the indications and safety of NOSES for colorectal cancer. After 2015, research related to NOSES gradually increased, filling the gaps in the research on indications and safety. Subsequently, the field of colorectal cancer NOSES entered the phase of guideline standardization. In 2015, a clinical trial investigated the short-term outcomes of patients with rectal cancer undergoing NOSES, considering NOSES a feasible and safe technique with shorter operative time and lower early readmission rates (20). In the same year, a review also elaborated on the reliability of NOSES for the treatment of early rectal cancer. This study ranks fourth in terms of total citations in the field of colorectal cancer NOSES (21). Considering the progress in research on NOSES for colorectal cancer, the 2017 ESMO guidelines recommended the application of NOSES in low rectal cancer (22). In terms of safety, a large-scale global prospective study in 2019 explored the risk factors for anastomotic leakage after NOSES, including smoking, obesity, and prolonged operative time (23). In this phase, a study published in 2020 by WASMUTH HH in *BRIT J SURG* highlighted the high rate of anastomotic leakage after NOSES, as well as unfavorable local recurrence rates and growth patterns (24). This led to the first comprehensive suspension of NOSES for rectal cancer in Norway, raising widespread concerns about the safety of NOSES for colorectal cancer. However, recent studies have also demonstrated that NOSES yields satisfactory short-term and long-term outcomes, thereby confirming its reliability (25–28). It is essential to continue monitoring its applicability and safety. With the advancement of artificial intelligence-related fields, research on NOSES continued to be updated. After 2020, research in this field has tended towards the use of new technologies such as robotics, with multidisciplinary integration promoting the development of NOSES. Structured training and robotic assistance may become important pathways to improve the quality of NOSES (29, 30).

### The characteristic of NOSES in terms of painlessness, asepsis, and oncological safety principles

The field of minimally invasive surgery for colorectal tumors is undergoing a conceptual revolution, shifting from minimally invasive to non-invasive approaches. NOSES, an emerging technology that is nearly non-invasive and does not depend on abdominal wall incisions, has emerged as a research hotspot in colorectal tumor surgery. It emphasizes three key aspects: painlessness, asepsis, and tumor-free techniques (10, 31, 32). Compared with conventional laparoscopic specimen extraction surgery, NOSES surgery results in less intraoperative bleeding and faster postoperative recovery (33). Additionally, it reduces the incidence of conversion surgeries, which not only refers to the conversion to open surgery but also encompasses any alteration to the initially planned procedure (34). This effectively encapsulates the technical superiority of the “bottom-to-up” approach inherent to NOSES. In recent years, the combination of NOSES with artificial intelligence and robotic surgery has amplified its advantages in minimally invasive treatment (35, 36).

Contemporary healthcare, increasingly centered on patient-reported outcomes, places a heightened emphasis on patient experience. The detrimental impact of postoperative pain on patients is substantial. NOSES confers a distinct advantage by precluding the necessity for incisions rather than merely minimizing their size, thereby exerting a significant influence on mitigating postoperative pain (37). Patients undergoing NOSES require less postoperative analgesic medication compared with those undergoing traditional laparoscopic surgery. Moreover, patients in the NOSES group have lower postoperative VAS scores, highlighting the superior efficacy of NOSES in reducing postoperative pain (38). A clinical study in 2019 demonstrated that patients with distal rectal cancer treated with NOSES had lower postoperative pain and anxiety scores compared with those treated with traditional laparoscopic surgery, elucidating the pronounced advantages of NOSES from the subjective perspective of patients (39). However, another study in the same year indicated that taTME might cause more severe postoperative perineal pain (40). Therefore, a comprehensive assessment of incisional pain and perineal pain has become a focus in the postoperative management of NOSES.

NOSES performs specimen extraction and gastrointestinal reconstruction through natural orifices, thereby avoiding the creation of abdominal wall incisions. This approach significantly reduces the incidence of incision-related complications, such as incisional infections (41, 42). A cohort study in 2024 indicated that NOSES can reduce the incidence of incisional infections and is associated with a higher level of safety (43). While NOSES eliminates the need for abdominal wall incisions, thereby removing the risk of abdominal wall incision infections, it necessitates the creation of an additional incision through a natural orifice (vagina, rectum) to remove the surgical specimen. On the other hand, NOSES requires the opening of the intestinal cavity within the abdomen to complete gastrointestinal reconstruction. If these procedures are not performed in a standardized manner, maintaining asepsis will face a significant challenge (44). Previous studies have indicated that among postoperative infections caused by NOSES, the risk of bacterial contamination via the colonic route is the highest (45). In a prospective study where peritoneal infections were cultured, 39% were identified as gastrointestinal flora (46). Ensuring meticulous bowel preparation, using a linear stapler to close the proximal and distal margins of the tumor, irrigating the rectal stump with diluted 1% povidone-iodine before opening it, and employing sterile protective drapes are all measures that can effectively reduce the incidence of infection (47). The research team of Linke demonstrated that after vaginal disinfection with povidone-iodine tablets and xylocaine, in NOSES procedures, only a minority of patients experienced microbial contamination of the peritoneal cavity, and no surgical site infections occurred postoperatively (48). Additionally, during NOSES, the rational use of protective sheaths and suction devices when extracting specimens can significantly reduce the infection rate, a conclusion that has been confirmed by randomized controlled trial (49). Professor Cai Jianchun’s team proposed that traditional soft protective sheaths lack supportive function and may damage the intestinal mucosa. Therefore, they invented the “Cai’s Trocar,” which reduces damage to the anus and effectively prevents peritoneal infection and tumor implantation (50, 51). Innovative surgical instruments and improved surgical techniques have facilitated progress in the asepsis and tumor-free aspects of NOSES.

In radical surgery for colorectal cancer, “tumor-free operation” is the most fundamental requirement. Compared with the minimally invasive nature of NOSES, tumor-free should have higher priority. The analysis of data from 66 registered units across 23 countries showed that NOSES has good oncological safety, low rate of involved margins, good specimen quality, and acceptable short-term patient outcomes (15). Particularly among individuals with higher BMI, NOSES confers the advantage of a lower risk of distal margin involvement (52). However, in the overall population, NOSES has a similar local recurrence rate to laparoscopic surgery. Larsen et al. reported potential risk factors, including incorrect dissection planes and suboptimal surgical techniques (53). While there is no significant difference in tumor-free pathological outcomes between NOSES and laparoscopic surgery, this may be related to the lack of technical proficiency and experience among NOSES surgeons (54). Therefore, with a well-defined learning curve, the advantages of NOSES may be enhanced, potentially bringing more benefits to the patient population for whom it is indicated.

### Future directions and societal impact

Compared with traditional reviews, bibliometric analysis has the characteristic of capturing thematic evolution, emerging trends, and underexplored areas, while providing a data-driven basis for topic selection and future research directions (55). Therefore, through bibliometric analysis, we objectively evaluated the current state of NOSES research to predict future research directions and societal impact. Reviewing previous studies, we found that there is an urgent need for large-scale, multicenter randomized controlled trials comparing NOSES with conventional laparoscopic surgery to obtain long-term oncological endpoints (5-year disease-free survival and overall survival). Furthermore, we advocate for the implementation of feasibility studies in low- and middle-income countries to address existing healthcare disparities between high-income countries and LMICs. On the other hand, keyword burst analysis indicates that with the application of artificial intelligence in the medical field, AI-assisted surgical planning may be a promising frontier for the development of NOSES. In terms of social impact, we emphasize the important value of patient reported outcomes in the field of NOSES, which can help elucidate how reduced postoperative pain and accelerated recovery directly improve patients’ reported quality of life. On the other hand, NOSES may lead to potential cost savings by reducing incision-related complications and shortening hospital stays.

### Strengths and limitations

This study has several strengths. First, this study is the first to comprehensively use three recognized bibliometric tools for bibliometric analysis of NOSES for colorectal cancer, with cross- comparison among different software. Additionally, this paper is helpful for researchers interested in this field, enhancing their understanding of the research frontier. Moreover, this study elaborates on the importance of structured training and robotic assistance, highlighting the trend of multidisciplinary integration. Ultimately, through high-quality scientific bibliometric analysis, this study provides references for improving the safety and reliability of NOSES.

This study acknowledges several limitations. First, as a bibliometric analysis, data processing based on software cannot fully replace systematic manual retrieval; nevertheless, it remains a reliable approach for large-scale literature analytics. Second, the exclusive use of the Web of Science Core Collection (WoSCC) is a common constraint in bibliometric studies. However, given WoSCC’s comprehensive coverage, analyses derived from it are scientifically sound and reliable. Finally, citation impact lag may result in high-quality, recently published studies being under-recognized.

## Conclusion

In summary, research on NOSES for colorectal cancer has garnered increasing attention over the past five years, underscoring the significance of this field. Using bibliometric and visualization methods, this study delineated the evolution of NOSES research over the past 15 years. Findings indicate that prior consensus statements and guidelines have standardized indications and safety, while future advances will likely arise through multidisciplinary integration. Building on these insights, investigators can better define future research priorities and optimize clinical management of colorectal cancer patients undergoing NOSES, ultimately improving outcomes.

## Data Availability

The original contributions presented in the study are included in the article/**Supplementary Material**. Further inquiries can be directed to the corresponding authors.
