# Supplementary material for: Multi-database bibliometric analysis and cross-database comparison of NOSES research in colorectal cancer: integrated evidence from WoSCC and PubMed
Source: Front Oncol. 2026 May 20;16:1803748. doi: 10.3389/fonc.2026.1803748 (PMC13229740; doi:10.3389/fonc.2026.1803748)
Supplement: Supplementary file 1 [file DataSheet1.docx]

**Supplementary Materials**

Owing to the limited scope of this article, we have relegated several significant tables to the supplementary materials. These supplementary materials serve to comprehensively delineate the current state of research in the field of colorectal cancer NOSES and to forecast future developmental trajectories.

Table 1Countries' Scientific Production in colorectal cancer NOSES.

| **Country** | **Freq** |
| --- | --- |
| CHINA | 273 |
| NETHERLANDS | 189 |
| USA | 148 |
| UK | 134 |
| ITALY | 118 |
| SPAIN | 77 |
| GERMANY | 72 |
| JAPAN | 64 |
| FRANCE | 48 |
| CANADA | 41 |
| DENMARK | 35 |
| SOUTH KOREA | 35 |
| NORWAY | 28 |
| BRAZIL | 23 |
| AUSTRALIA | 22 |
| BELGIUM | 19 |
| SINGAPORE | 17 |
| PORTUGAL | 14 |
| AUSTRIA | 13 |
| ISRAEL | 13 |
| SWEDEN | 12 |
| TURKEY | 11 |
| SWITZERLAND | 10 |
| POLAND | 9 |
| IRELAND | 7 |

Table 2 Most Cited Countries in colorectal cancer NOSES.

| **Country** | **TC** | **Average Article Citations** |
| --- | --- | --- |
| UNITED KINGDOM | 2306 | 100.30 |
| USA | 1606 | 24.30 |
| NETHERLANDS | 1599 | 55.10 |
| CHINA | 1191 | 10.80 |
| SPAIN | 1058 | 44.10 |
| ITALY | 710 | 25.40 |
| FRANCE | 632 | 31.60 |
| KOREA | 320 | 26.70 |
| GERMANY | 279 | 17.40 |
| NORWAY | 273 | 68.20 |
| DENMARK | 265 | 29.40 |
| JAPAN | 242 | 15.10 |
| BELGIUM | 216 | 43.20 |
| SWITZERLAND | 172 | 43.00 |
| CANADA | 100 | 11.10 |
| TURKEY | 82 | 11.70 |
| AUSTRALIA | 77 | 12.80 |
| BRAZIL | 70 | 14.00 |
| COLOMBIA | 65 | 65.00 |
| POLAND | 47 | 15.70 |
| IRELAND | 37 | 12.30 |
| SINGAPORE | 29 | 3.20 |
| JAMAICA | 25 | 25.00 |
| RUSSIA | 25 | 25.00 |
| CZECH REPUBLIC | 24 | 24.00 |

Table 3 Most Relevant Affiliations in colorectal cancer NOSES.

| **Affiliation** | **Articles** |
| --- | --- |
| CHINESE ACADEMY OF MEDICAL SCIENCES - PEKING UNION MEDICAL COLLEGE | 35 |
| UNIVERSITY OF AMSTERDAM | 29 |
| PEKING UNION MEDICAL COLLEGE | 27 |
| CANCER INSTITUTE AND HOSPITAL - CAMS | 26 |
| LEIDEN UNIVERSITY | 20 |
| LEIDEN UNIVERSITY MEDICAL CENTER (LUMC) | 20 |
| VRIJE UNIVERSITEIT AMSTERDAM | 19 |
| LANKENAU MEDICAL CENTER | 17 |
| SUN YAT SEN UNIVERSITY | 17 |
| ASSISTANCE PUBLIQUE HOPITAUX PARIS (APHP) | 14 |
| CENTRAL SOUTH UNIVERSITY | 14 |
| IMPERIAL COLLEGE LONDON | 14 |
| LEIDEN UNIVERSITY - EXCL LUMC | 14 |
| UNIVERSITY OF BARCELONA | 14 |
| ACADEMIC MEDICAL CENTER AMSTERDAM | 13 |
| AUTONOMOUS UNIVERSITY OF BARCELONA | 13 |
| CLEVELAND CLINIC FOUNDATION | 13 |
| HARBIN MEDICAL UNIVERSITY | 13 |
| UNIVERSITY OF BIRMINGHAM | 13 |
| ADVENTHEALTH | 12 |
| ADVENTIST HEALTH SERVICES | 12 |
| ERASMUS MC | 12 |
| ERASMUS UNIVERSITY ROTTERDAM | 12 |
| UNICANCER | 12 |
| UNIVERSITY OF COPENHAGEN | 12 |

Table 4 Author basic information in colorectal cancer NOSES.

| **Author** | **H index** | **G index** | **M index** | **TC** | **NP** | **PY start** |
| --- | --- | --- | --- | --- | --- | --- |
| HOMPES R | 11 | 17 | 1 | 848 | 17 | 2015 |
| LACY AM | 9 | 10 | 0.563 | 1052 | 10 | 2010 |
| TANIS PJ | 9 | 11 | 0.75 | 829 | 11 | 2014 |
| PENNA M | 8 | 8 | 0.8 | 642 | 8 | 2016 |
| SIETSES C | 8 | 8 | 0.615 | 617 | 8 | 2013 |
| TUYNMAN JB | 8 | 9 | 0.8 | 487 | 9 | 2016 |
| WANG XS | 8 | 16 | 1 | 279 | 16 | 2018 |
| ATALLAH S | 7 | 7 | 0.583 | 273 | 7 | 2014 |
| CUNNINGHAM C | 7 | 7 | 0.636 | 221 | 7 | 2015 |
| SYLLA P | 7 | 9 | 0.438 | 798 | 9 | 2010 |
| DOORNEBOSCH PG | 6 | 7 | 0.375 | 431 | 7 | 2010 |
| MARESCAUX J | 6 | 6 | 0.4 | 218 | 6 | 2011 |
| MARIJNEN CAM | 6 | 7 | 0.429 | 762 | 7 | 2012 |
| MARKS JH | 6 | 9 | 0.6 | 105 | 9 | 2016 |
| WANG GY | 6 | 11 | 0.857 | 218 | 11 | 2019 |
| ZHOU HT | 6 | 9 | 0.5 | 226 | 9 | 2014 |
| BEMELMAN WA | 5 | 6 | 0.5 | 241 | 6 | 2016 |
| DE WILT JHW | 5 | 5 | 0.313 | 398 | 5 | 2010 |
| KAYAALP C | 5 | 8 | 0.417 | 203 | 8 | 2014 |
| MORTENSEN NJ | 5 | 5 | 0.455 | 387 | 5 | 2015 |
| YAO HL | 5 | 6 | 0.714 | 160 | 6 | 2019 |
| BACH SP | 4 | 4 | 0.364 | 109 | 4 | 2015 |
| BROWN G | 4 | 4 | 0.308 | 1931 | 4 | 2013 |
| BULUT O | 4 | 5 | 0.308 | 115 | 5 | 2013 |
| CHEN WTL | 4 | 5 | 0.444 | 97 | 5 | 2017 |

Table 5 Table of the international influence of journals in colorectal cancer NOSES.

| **Source** | **H index** | **G index** | **M index** | **TC** | **NP** | **PY start** |
| --- | --- | --- | --- | --- | --- | --- |
| SURGICAL ENDOSCOPY AND OTHER INTERVENTIONAL TECHNIQUES | 25 | 46 | 1.563 | 2316 | 46 | 2010 |
| TECHNIQUES IN COLOPROCTOLOGY | 18 | 29 | 1.125 | 901 | 35 | 2010 |
| COLORECTAL DISEASE | 16 | 25 | 1.067 | 633 | 29 | 2011 |
| DISEASES OF THE COLON & RECTUM | 14 | 25 | 0.875 | 670 | 25 | 2010 |
| WORLD JOURNAL OF GASTROENTEROLOGY | 9 | 11 | 0.6 | 318 | 11 | 2011 |
| INTERNATIONAL JOURNAL OF COLORECTAL DISEASE | 7 | 14 | 0.438 | 198 | 14 | 2010 |
| BRITISH JOURNAL OF SURGERY | 6 | 6 | 0.462 | 514 | 6 | 2013 |
| CLINICS IN COLON AND RECTAL SURGERY | 5 | 8 | 0.455 | 66 | 9 | 2015 |
| JOURNAL OF LAPAROENDOSCOPIC & ADVANCED SURGICAL TECHNIQUES | 5 | 10 | 0.333 | 100 | 13 | 2011 |
| AMERICAN JOURNAL OF SURGERY | 4 | 4 | 0.571 | 63 | 4 | 2019 |
| ANNALS OF SURGERY | 4 | 5 | 0.308 | 586 | 5 | 2013 |
| ANNALS OF SURGICAL ONCOLOGY | 4 | 5 | 0.25 | 243 | 5 | 2010 |
| ANNALS OF SURGICAL TREATMENT AND RESEARCH | 4 | 6 | 0.333 | 56 | 6 | 2014 |
| BMJ OPEN | 4 | 4 | 0.444 | 103 | 4 | 2017 |
| EJSO | 4 | 7 | 0.333 | 180 | 7 | 2014 |
| FRONTIERS IN ONCOLOGY | 4 | 6 | 0.667 | 46 | 9 | 2020 |
| JOURNAL OF SURGICAL ONCOLOGY | 4 | 5 | 0.25 | 59 | 5 | 2010 |
| SURGICAL INNOVATION | 4 | 7 | 0.286 | 111 | 7 | 2012 |
| SURGICAL LAPAROSCOPY ENDOSCOPY & PERCUTANEOUS TECHNIQUES | 4 | 7 | 0.267 | 59 | 7 | 2011 |
| SURGICAL ONCOLOGY-OXFORD | 4 | 5 | 1 | 120 | 5 | 2022 |
| WORLD JOURNAL OF SURGERY | 4 | 6 | 0.267 | 262 | 6 | 2011 |
| ANNALI ITALIANI DI CHIRURGIA | 3 | 3 | 0.273 | 21 | 3 | 2015 |
| ASIAN JOURNAL OF SURGERY | 3 | 3 | 0.429 | 12 | 4 | 2019 |
| BMC SURGERY | 3 | 3 | 0.6 | 18 | 7 | 2021 |
| CANCER MANAGEMENT AND RESEARCH | 3 | 3 | 0.375 | 80 | 3 | 2018 |

Table 6 Core journals by Bradford's Law in colorectal cancer NOSES.

| **Source** | **Rank** | **Freq** | **cumFreq** | **Zone** |
| --- | --- | --- | --- | --- |
| SURGICAL ENDOSCOPY AND OTHER INTERVENTIONAL TECHNIQUES | 1 | 46 | 46 | Zone 1 |
| TECHNIQUES IN COLOPROCTOLOGY | 2 | 35 | 81 | Zone 1 |
| COLORECTAL DISEASE | 3 | 29 | 110 | Zone 1 |
| DISEASES OF THE COLON & RECTUM | 4 | 25 | 135 | Zone 1 |
| INTERNATIONAL JOURNAL OF COLORECTAL DISEASE | 5 | 14 | 149 | Zone 1 |
| JOURNAL OF LAPAROENDOSCOPIC & ADVANCED SURGICAL TECHNIQUES | 6 | 13 | 162 | Zone 2 |
| WORLD JOURNAL OF GASTROENTEROLOGY | 7 | 11 | 173 | Zone 2 |
| CLINICS IN COLON AND RECTAL SURGERY | 8 | 9 | 182 | Zone 2 |
| FRONTIERS IN ONCOLOGY | 9 | 9 | 191 | Zone 2 |
| BMC SURGERY | 10 | 7 | 198 | Zone 2 |
| EJSO | 11 | 7 | 205 | Zone 2 |
| SURGICAL INNOVATION | 12 | 7 | 212 | Zone 2 |
| SURGICAL LAPAROSCOPY ENDOSCOPY & PERCUTANEOUS TECHNIQUES | 13 | 7 | 219 | Zone 2 |
| ANNALS OF SURGICAL TREATMENT AND RESEARCH | 14 | 6 | 225 | Zone 2 |
| BRITISH JOURNAL OF SURGERY | 15 | 6 | 231 | Zone 2 |
| FRONTIERS IN SURGERY | 16 | 6 | 237 | Zone 2 |
| MINERVA CHIRURGICA | 17 | 6 | 243 | Zone 2 |
| VIDEOSURGERY AND OTHER MINIINVASIVE TECHNIQUES | 18 | 6 | 249 | Zone 2 |
| WORLD JOURNAL OF SURGERY | 19 | 6 | 255 | Zone 2 |
| ANNALS OF SURGERY | 20 | 5 | 260 | Zone 2 |
| ANNALS OF SURGICAL ONCOLOGY | 21 | 5 | 265 | Zone 2 |
| JOURNAL OF SURGICAL ONCOLOGY | 22 | 5 | 270 | Zone 2 |
| MEDICINE | 23 | 5 | 275 | Zone 2 |
| SURGICAL ONCOLOGY-OXFORD | 24 | 5 | 280 | Zone 2 |
| UPDATES IN SURGERY | 25 | 5 | 285 | Zone 2 |

Table 7 Most local cited documents in colorectal cancer NOSES.

| **Document** | **Year** | **Local Citations** | **Global Citations** | **LC/GC Ratio (%)** | **Normalized Local Citations** | **Normalized Global Citations** |
| --- | --- | --- | --- | --- | --- | --- |
| SYLLA P, 2010, SURG ENDOSC | 2010 | 87 | 575 | 15.13 | 9.98 | 8.97 |
| WOLTHUIS AM, 2015, BRIT J SURG | 2015 | 39 | 122 | 31.97 | 8.63 | 2.72 |
| GUAN X, 2019, GASTROENTEROL REP | 2019 | 39 | 123 | 31.71 | 8.03 | 3.67 |
| PENNA M, 2019, ANN SURG | 2019 | 34 | 248 | 13.71 | 7.00 | 7.40 |
| FRANKLIN ME, 2013, TECH COLOPROCTOL | 2013 | 25 | 75 | 33.33 | 3.32 | 1.56 |
| FERNÁNDEZ-HEVIA M, 2015, ANN SURG | 2015 | 25 | 218 | 11.47 | 5.53 | 4.86 |
| WASMUTH HH, 2020, BRIT J SURG | 2020 | 25 | 194 | 12.89 | 7.69 | 10.13 |
| FRANCIS N, 2017, SURG ENDOSC | 2017 | 21 | 124 | 16.94 | 5.41 | 1.84 |
| ADAMINA M, 2018, SURG ENDOSC | 2018 | 20 | 120 | 16.67 | 7.87 | 5.42 |
| KARAGUL S, 2017, TECH COLOPROCTOL | 2017 | 19 | 34 | 55.88 | 4.89 | 0.51 |
| LEUNG ALH, 2013, WORLD J SURG | 2013 | 18 | 69 | 26.09 | 2.39 | 1.43 |
| VELTHUIS S, 2014, SURG ENDOSC | 2014 | 18 | 133 | 13.53 | 5.63 | 3.20 |
| HELBACH MV, 2016, SURG ENDOSC | 2016 | 18 | 112 | 16.07 | 3.50 | 2.82 |
| SAURABH B, 2017, DIS COLON RECTUM | 2017 | 18 | 56 | 32.14 | 4.64 | 0.83 |
| MA B, 2015, INT J COLORECTAL DIS | 2015 | 17 | 57 | 29.82 | 3.76 | 1.27 |
| ARROYAVE MC, 2017, EJSO-EUR J SURG ONC | 2017 | 17 | 65 | 26.15 | 4.38 | 0.97 |
| LEROY J, 2013, JAMA SURG | 2013 | 16 | 101 | 15.84 | 2.13 | 2.09 |
| MARKS J, 2010, SURG ENDOSC | 2010 | 15 | 67 | 22.39 | 1.72 | 1.05 |
| AWAD ZT, 2014, SURG ENDOSC | 2014 | 15 | 38 | 39.47 | 4.69 | 0.91 |
| ZHOU SC, 2019, CANCER MANAG RES | 2019 | 15 | 32 | 46.88 | 3.09 | 0.95 |
| SYLLA P, 2013, SURG ENDOSC | 2013 | 14 | 89 | 15.73 | 1.86 | 1.85 |
| MCLEMORE EC, 2016, SURG ENDOSC | 2016 | 14 | 59 | 23.73 | 2.73 | 1.49 |
| HOL JC, 2019, TECH COLOPROCTOL | 2019 | 14 | 89 | 15.73 | 2.88 | 2.66 |
| DOORNEBOSCH PG, 2010, DIS COLON RECTUM | 2010 | 13 | 93 | 13.98 | 1.49 | 1.45 |
| ZORRON R, 2012, SURG INNOV | 2012 | 13 | 64 | 20.31 | 7.15 | 3.50 |

Table 8 Most frequent keywords in colorectal cancer NOSES.

| **Keywords** | **Occurrences** |
| --- | --- |
| total mesorectal excision | 167 |
| surgery | 108 |
| resection | 83 |
| colorectal-cancer | 80 |
| rectal-cancer | 76 |
| outcomes | 67 |
| trial | 59 |
| transanal endoscopic microsurgery | 57 |
| colectomy | 48 |
| risk-factors | 48 |
| low anterior resection | 42 |
| quality-of-life | 42 |
| cancer | 40 |
| anterior resection | 35 |
| local excision | 32 |
| minimally invasive surgery | 27 |
| multicenter | 27 |
| pathological outcomes | 27 |
| short-term outcomes | 27 |
| preoperative radiotherapy | 26 |
| colorectal surgery | 25 |
| metaanalysis | 24 |
| local recurrence | 23 |
| survival | 23 |
| colon | 22 |

Table 9 Surgical Techniques and Classification of Natural Orifice Specimen Extraction Surgery for Colorectal Cancer^[1]^.

| **Classification (CRC-NOSES)** | **Modus operandi** | **Tumor location** | **Orifice** |
| --- | --- | --- | --- |
| I | Laparoscopic lower rectal cancer resection with transanal specimen extraction | Lower rectum | Anus |
| II | Laparoscopic middle rectal cancer resection with transanal specimen extraction | Middle rectum | Anus |
| III | Laparoscopic middle rectal cancer resection with transvaginal specimen extraction | Middle rectum | Vagina |
| IV | Laparoscopic upper rectal cancer resection with transanal specimen extraction | Upper rectum/distal sigmoid colon | Anus |
| V | Laparoscopic upper rectal cancer resection with transvaginal specimen extraction | Upper rectum/distal sigmoid colon | Vagina |
| VI | Laparoscopic upper rectal cancer resection with transanal specimen extraction | Left colon/proximal sigmoid colon | Anus |
| VII | Laparoscopic left colectomy with transvaginal specimen extraction | Left colon/proximal sigmoid colon | Vagina |
| VIII | Laparoscopic right colectomy with transvaginal specimen extraction | Right colon | Vagina |
| IX | Laparoscopic total colectomy with transanal specimen extraction | Total colon | Anus |
| X | Laparoscopic total colectomy with transvaginal specimen extraction | Total colon | Vagina |


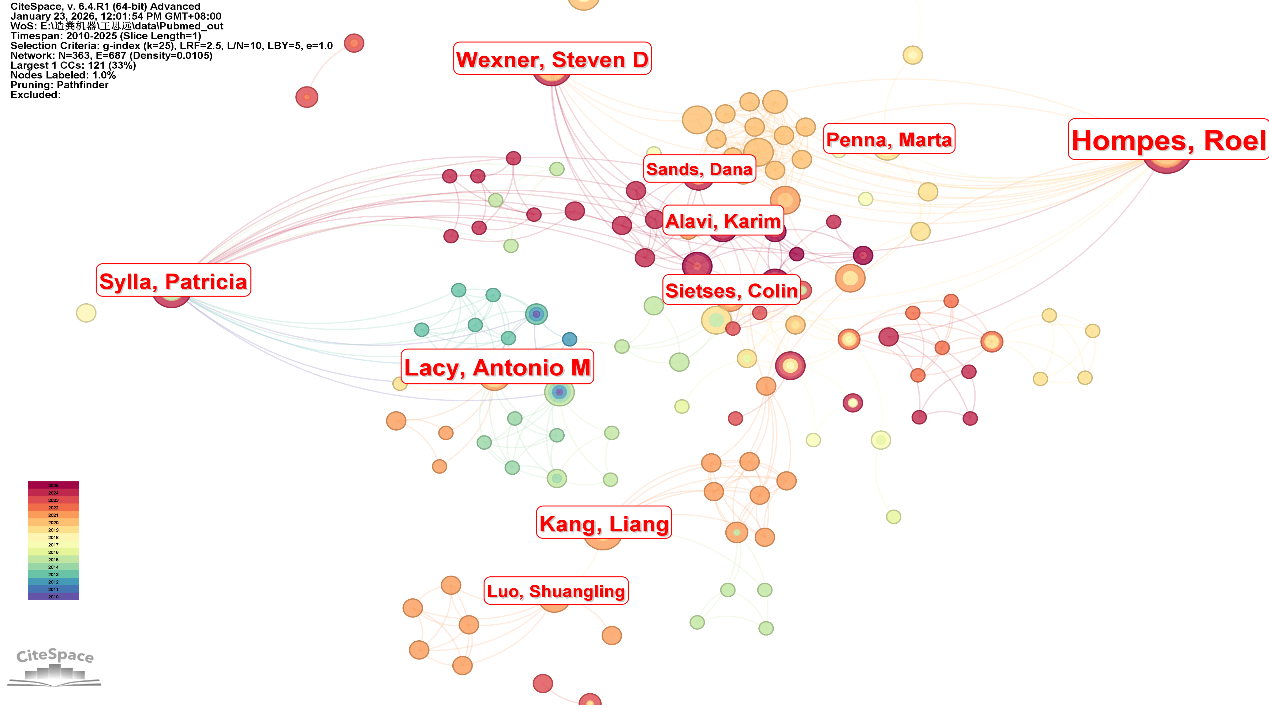


Figure 1 Author Collaboration Network Map Based on the PubMed Database.


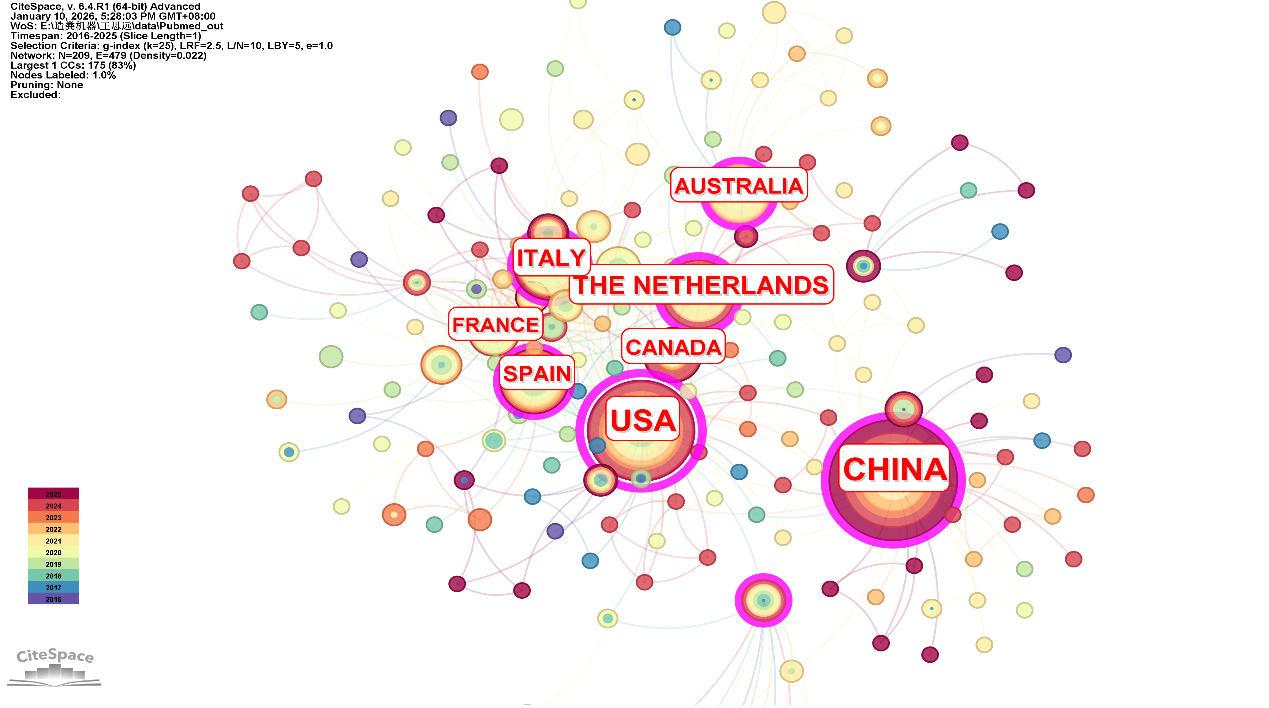


Figure 2 Country Collaboration Network Map Based on the PubMed Database.


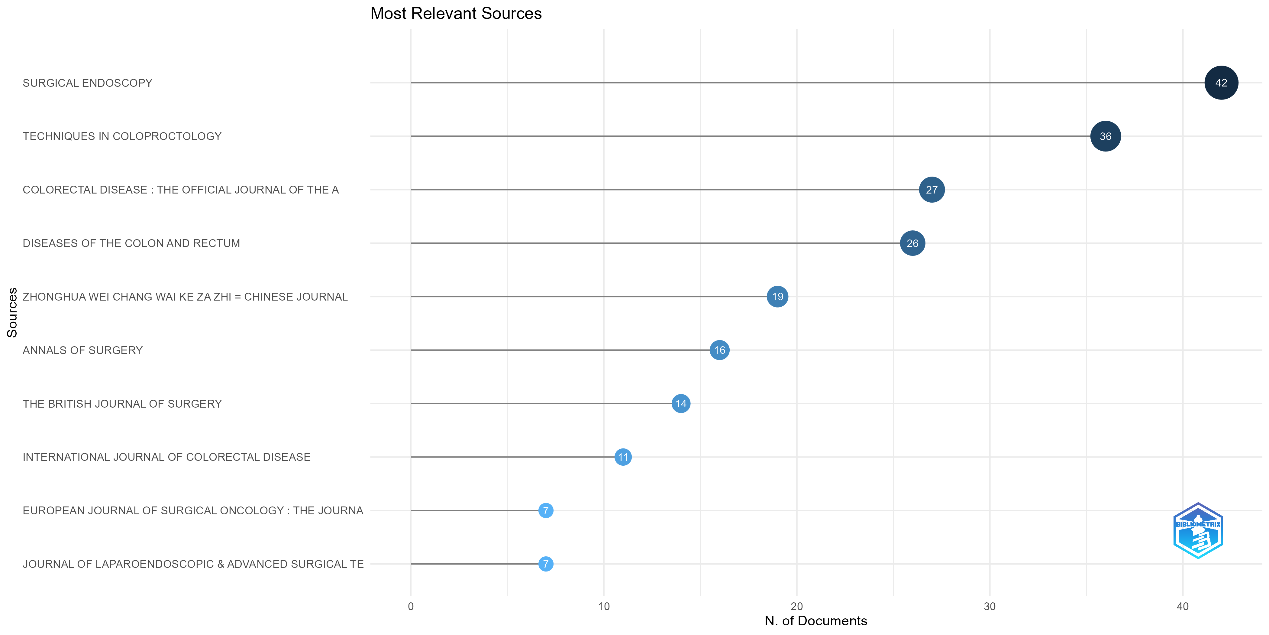


Figure 3 Journal Ranking Map Based on the PubMed Database.


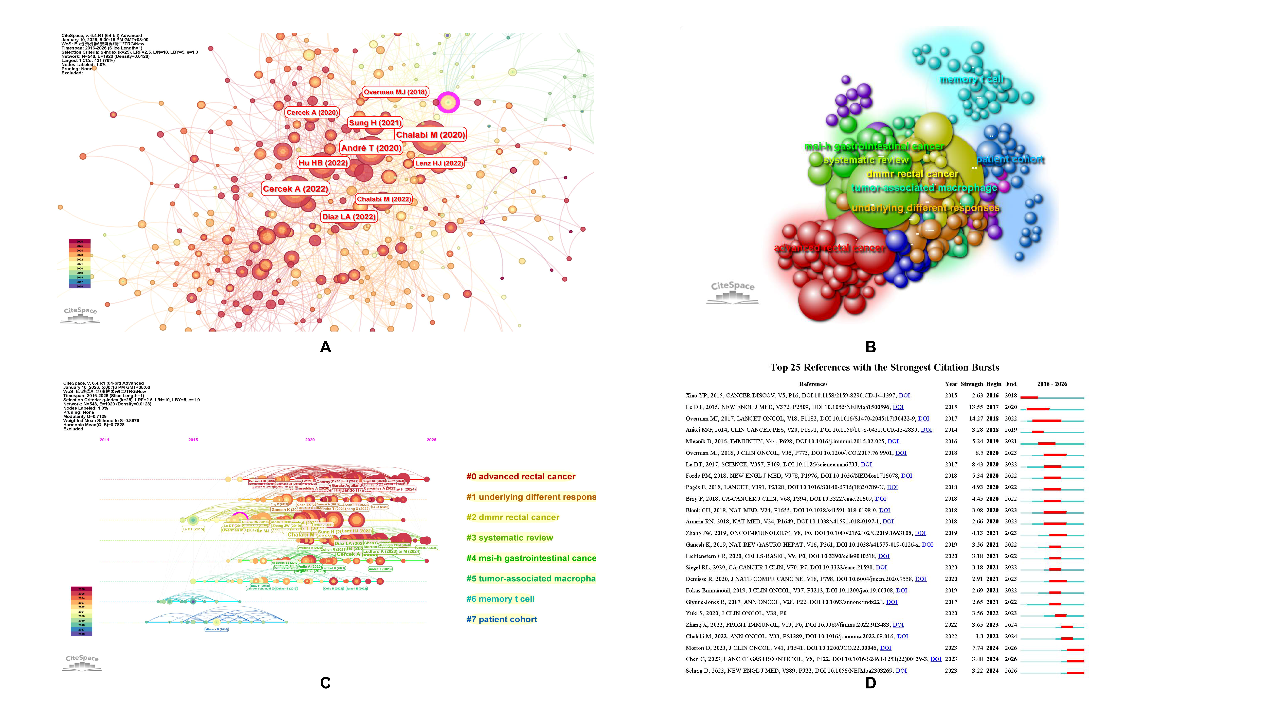


Figure 4 Co cited Literature Analysis Based on the PubMed Database. (A) Co cited Literature Interaction Map. (B) Co cited Literature Label Clustering Analysis. (C) Co-cited Literature Temporal Analysis Map. (D) Co-cited Literature Burst Detection Map.


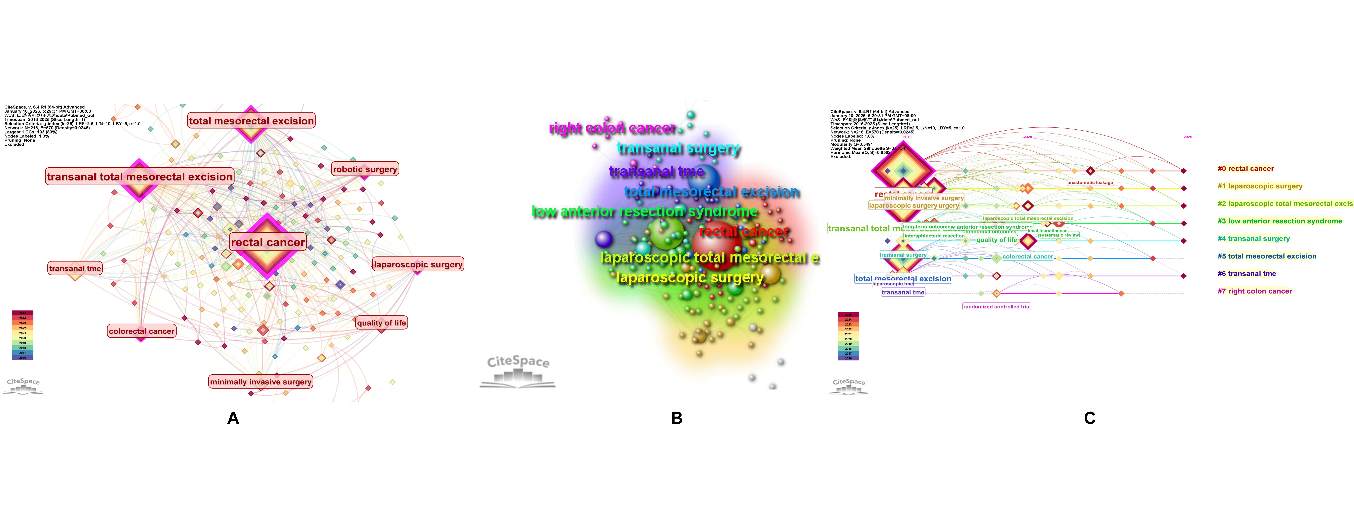


Figure 5 Keyword Analysis Based on the PubMed Database. (A) Keyword Interaction Map. (B) Co-cited Literature Label Clustering Analysis. (C) Co-cited Literature Temporal Analysis Map.

**References**

[1] Guan X, Liu Z, Longo A, et al. International consensus on natural orifice specimen extraction surgery (NOSES) for colorectal cancer. Gastroenterol Rep (Oxf). 2019. 7(1): 24-31.
